# Polydimethylsiloxane Elastomers Filled with Rod-Like α-MnO_2_ Nanoparticles: An Interplay of Structure and Electrorheological Performance

**DOI:** 10.3390/polym12122810

**Published:** 2020-11-27

**Authors:** Alexander V. Agafonov, Anton S. Kraev, Anastasia A. Egorova, Alexander E. Baranchikov, Sergey A. Kozyukhin, Vladimir K. Ivanov

**Affiliations:** 1 Krestov Institute of Solution Chemistry of the Russian Academy of Sciences, 153045 Ivanovo, Russia; ava@isc-ras.ru (A.V.A.); a.s.kraev@mail.ru (A.S.K.); 2 Department of Chemistry, National Research Tomsk State University, 634050 Tomsk, Russia; 3 Kurnakov Institute of General and Inorganic Chemistry of the Russian Academy of Sciences, 119991 Moscow, Russia; ana.egorova13@ya.ru (A.A.E.); a.baranchikov@yandex.ru (A.E.B.); sergkoz@igic.ras.ru (S.A.K.); 4 Department of Chemistry, National Research University Higher School of Economics, 101000 Moscow, Russia

**Keywords:** nanocomposites, electrorheological elastomers, smart materials, Payne effect, vulcanisation, manganese dioxide

## Abstract

For the first time, electroactive nanocomposite elastomers based on polydimethylsiloxane and filled with rod-like α-MnO_2_ nanoparticles have been obtained. The curing of the filled elastomer in an electric field, resulting in the ordering of the α-MnO_2_ particles, had a significant effect on the degree of polymer crosslinking, as well as on the electrorheological characteristics of the nanocomposites obtained through this process, namely the values of the storage and loss moduli. The dielectric spectra of filled elastomers in the frequency range 25–10^6^ Hz were analysed in terms of interfacial relaxation processes. It has been shown, for the first time, that the application of an electric field leads to a decrease in the value of the Payne effect in composite elastomers. Analysis of the rheological effect in the obtained materials has demonstrated the possibility of designing highly efficient electrorheological elastomers that change their elastic properties by 4.3 times in electric fields of up to 2 kV/mm.

## 1. Introduction

Electrorheological and magnetorheological elastomers are elastic polymer matrices filled with semiconductor or ferromagnetic nano- or microparticles that are highly polarisable in electromagnetic fields. Optimism for the practical use of such polymer composites is encouraged by the possibility of a controlled and reversible change in their rheological properties when an external electric or magnetic field is applied [1,2,3]. The design of new high-performance smart materials that change their elastic properties in an electric field is currently one of the challenges for materials science [4,5,6].

The magnitude of the rheological response of a material to an external field depends on many factors, including the composition of the polymer matrix and filler, as well as the concentration and size of its particles. One key factor is the spatial distribution of filler particles in the volume of the elastomer [7,8]. Current progress in the development of new highly active electro- or magnetorheological materials is largely associated with the production of new types of fillers for elastomers, which provide the greatest adaptive response to the external field. Nevertheless, in terms of the efficiency of converting the energy of the electromagnetic field into mechanical energy, electrorheological elastomers are still significantly inferior to magnetorheological elastomeric composites, and the search for new elastomeric materials with high electrorheological efficiency is of high scientific and practical interest [6]. Both inorganic (e.g., lead zirconate-titanate [9]) and polymeric (polythiophene [10], polydiphenylamine [11], poly(p-phenylene) [12], polyaniline [13,14]) particles have been proposed as promising fillers of electrorheological elastomers. The obtained electroactive composite elastomers have been shown to exhibit high storage modulus sensitivity (up to ~100%) in electric fields up to 2 kV/mm [12].

Recent progress in the engineering of electrorheological fluids could represent a strong basis for the design of efficient elastomeric composites [15,16]. The key parameters of composition and structure that determine the high electrorheological effect in electrorheological materials are the dielectric constant, the dielectric loss tangent, the electrical conductivity of the dispersed phase and the dispersion medium, the dielectric relaxation time [5,6,17,18,19,20,21,22], the concentration of particles of the dispersed phase and the nature of its spatial distribution, and the chemical composition of the surface of the dispersed phase particles, which determine the force of interaction between the filler particles [23,24,25,26].

The rigid structure of the polymer framework of electrorheological elastomers opens up new possibilities for increasing electrorheological efficiency by forming ordered structures from filler particles in a polymer matrix. For this, a liquid polymer containing filler particles is subjected to a static magnetic or electric field. As a result of polarisation interactions, the filler particles are arranged in chain structures. The electrorheological liquid structured in this way is subjected to vulcanisation, which makes it possible to fix the relative position of the dispersed phase particles [27,28].

An important consequence of the immobilisation of filler particles in the bulk of electrorheological elastomers is that such elastomers are devoid of one of the main disadvantages of electrorheological fluids—the sedimentation of dispersed phase particles, which leads to a gradual loss of the material’s functional properties. In this regard, dispersed materials with a relatively high density can be used as fillers for electrorheological elastomers. Note that high-density dispersed materials can provide a high electrorheological response: electrorheological fluids based on tungsten trioxide (7.4 g/cm^3^) [29], cerium dioxide (7.2 g/cm^3^) [30,31], bismuth sesquioxide (8.6 g/cm^3^) [32] and bismuth ferrites (6.5–8.4 g/cm^3^) [33,34] have been obtained previously, demonstrating high values of electrorheological response in static electric fields up to 5 kV/mm. At the same time, the introduction of surfactants or the use of highly concentrated suspensions (up to 60, and even 80, wt.% of the dispersed phase) was required to achieve high sedimentation stability of these materials.

Manganese dioxide and, specifically, one of its polymorphic modifications, α-MnO_2_, is a promising filler for electrorheological elastomers. The dielectric response of this compound is almost temperature independent, having a very high dielectric constant and low dielectric losses, which is typical of colossal dielectrics [35]. The possibility of manganese dioxide’s application for the manufacture of electrorheological elastomers is confirmed by the high electrorheological response of the corresponding electrorheological fluids [36]. At the same time, the high dielectric constant and low dielectric loss tangent of α-MnO_2_ make it possible to achieve high functional characteristics of electrorheological fluids with a sufficiently low filler content (up to 20 wt.%). From the point of view of creating electrorheological elastomeric materials, a low filler concentration ensures the low rigidity and high resilient-elastic properties of the composite, while high dielectric characteristics of the filler cause a high electrorheological effect. The rod-like shape of α-MnO_2_ nanoparticles can play an important role in the electrorheological performance of elastomers cured in an electric field. Presumably, in such elastomers, α-MnO_2_ particles will be oriented along the electric field, thus resulting in higher rigidity of the entire material.

The aim of the current work was to obtain novel electrorheological elastomers based on α-MnO_2_ nanoparticles and polydimethylsiloxane, as well as to analyse their electrorheological and dielectric properties.

## 2. Materials and Methods

### 2.1. Synthesis of Manganese Dioxide Nanorods (α-MnO_2_)

α-MnO_2_ powder was synthesised, based on the modified protocol reported elsewhere [37,38], using potassium permanganate (Khimmed, Moscow, Russia, PO0330), manganese(II) sulfate pentahydrate (Khimmed, Moscow, Russia, analytical grade, GOST 435-77) and distilled water as starting materials. The composition of MnSO_4_·5H_2_O was refined by gravimetric analysis before the synthesis. To synthesise manganese dioxide, 0.790 g of KMnO_4_ was dissolved in 70 mL of distilled water, 0.482 g of MnSO_4_·5H_2_O was added to the resulting solution and the mixture was stirred for 10 min. The mixed solution was transferred to a 100 mL Berghof DAP-100 Teflon autoclave (filled to ~70%). Hydrothermal treatment was carried out at 140 °C for 18 h, after which the autoclave was cooled in air and opened. The resulting precipitate was separated by filtration, washed with distilled water to pH 7 and dried at 100 °C for 9 h.

### 2.2. Preparation of Elastomeric Composites

Liquid siloxane rubber with terminal silanol groups (Viksint compound PK-68A TU 38.103508-81, degree of polymerisation *n* = 100–5000, OOO NPP Khimprom, Yaroslavl, Russia) was used as a starting material for obtaining the elastomeric matrix. The choice of siloxane rubbers as a basis for electrorheological composites was due to their wide range of operating temperatures and high modulus of elasticity [39]. To prepare filled elastomers with a dispersed phase content of 30 wt.% (9.4 vol.%), a weighed portion of α-MnO_2_ was mixed with an aliquot of organosilicon fluid. Then, the resulting mixture was stirred for an hour, with an overhead stirrer, at 500 rpm, after which 3 wt.% of the polymerisation catalyst was added (aminopropyltriethoxylane solution in tetraethoxysilane, mass ratio 1:4) [40], followed by stirring for an additional 30 min. The resulting suspension was evacuated (10^−2^ atm) to remove air bubbles. The mixture was cured in a cylindrical polymethyl methacrylate mould with a diameter of 20 mm, with flat stainless steel lids serving as electrodes. The distance between the electrodes was 2.5 mm. The samples were cured at room temperature without applying an electric voltage between the electrodes, or in an electric field of 2 kV/mm. Hereafter, the composite elastomer obtained in the absence of an electric field is designated as MnO_2_-0, while the sample obtained in an electric field is designated as MnO_2_-E.

### 2.3. Evaluation of the Degree of Crosslinking of the Elastomer

The swelling degree of the elastomer in toluene was used to estimate the swelling ratio [41]. Toluene ensures a very high swelling ratio of polydimethylsiloxane which is governed by the degree of polymer crosslinking only [42,43]. The samples of composite elastomers were immersed in toluene for 48 h at room temperature, with periodic stirring. During ageing, toluene was changed twice, at regular intervals. After ageing, the samples were removed from the toluene and the excess liquid was removed with filter paper and weighed, after which they were dried at 50 °C in a drying cabinet, under vacuum conditions (10^−2^ atm), to a constant weight and reweighed. When calculating the swelling ratio (*β*), gel fraction (*GF*) and uncured polymer content ($\omega_{c}$), the mass of manganese dioxide contained in the composite elastomer was taken into account. The calculations were carried out according to the formulas [44]:

(1) $$GF=\frac{m_{t}}{m_{0}}\cdot 100\%,$$

(2) $$\beta =\frac{m_{н}-m_{t}}{m_{t}}\cdot 100\%,$$

(3) $$\omega_{c}=\frac{m_{0}-m_{t}}{m_{0}}\cdot 100\%,$$

where $m_{0}$—the initial mass of the composite elastomer; $m_{н}$—the mass of swollen composite elastomer; $m_{t}$—the mass of the elastomer after drying. The Flory–Rehner equation was used to estimate the degree of crosslinking (ν) [45]:

(4) $$\nu =-\frac{\ln\left(1-\vartheta_{2m}\right)+\vartheta_{2m}+\chi_{12}\vartheta_{2m}^{2}}{V_{1}\cdot \left(\vartheta_{2m}^{\frac{1}{3}}-\frac{\vartheta_{2m}}{2}\right)},$$

where $V_{1}$—the molar volume of solvent (106.5 cm^3^/mol for toluene); $\vartheta_{2m}$—the molar fraction of the polymer in an equilibrium swelling state; $\chi_{12}$—the Flory-Higgins coefficient, characterising the interaction of polymer and solvent and being equal to 0.393 for the system analysed [46,47]. The calculation results are shown in Table 1.

### 2.4. Methods of Analysis

Powder X-ray diffraction (XRD) was performed using a Bruker D8 Advance diffractometer (Karlsruhe, Germany) (CuKα-radiation) in the range of 5–80°2θ, with a step of 0.02°2θ and acquisition duration of 0.3 s/step. Scanning electron microscopy (SEM) was carried out using a Carl Zeiss NVision 40 (Oberkochen, Germany) high-resolution electron microscope at 7 kV accelerating voltage.

The dependences of the dielectric constant and dielectric loss tangent of composite elastomers on the frequency of the electric field were measured in a capacitor-type cell with spring-loaded disk plane-parallel electrodes made of polished stainless steel, using a Solartron SI 1260 Impedance/Gain-Phase analyser (Farnborough, UK) at 1 V.

For electrorheological measurements, we used a rheometer operating in the controlled shear deformation mode, with a stepper motor with a controlled rotation speed and a torque measuring system. A voltage (up to 5.0 kV) was applied between the upper movable electrode and the lower fixed electrode connected to a strain gauge. The shear stress was measured every 10 s, at a rotation speed of 0.05 rad/s, until the shearing angle reached 0.192 rad. After the measurement, the rheometer plate was returned to its starting position. The absence of the electrode slip relative to the sample surface was checked by the coincidence of marks applied on the side faces of the electrodes and the elastomer sample.

## 3. Results and Discussion

**Figure 1:**
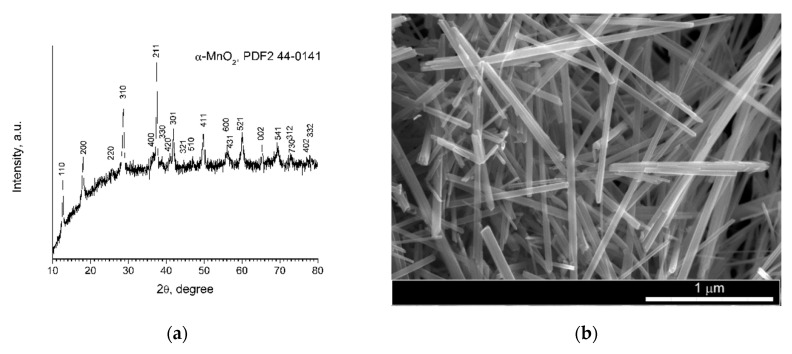
Results of (**a**) X-ray powder diffraction (XRD) and (**b**) SEM of α-MnO_2_ powder used as a filler for polydimethylsiloxane elastomer.

According to powder X-ray diffraction data, as a result of the hydrothermal treatment of a mixed solution of potassium permanganate and manganese(II) sulfate, nanocrystalline manganese dioxide (α-MnO_2_, *I*4/*m* space group) was obtained. The formation of nanocrystalline α-MnO_2_ under the chosen conditions of hydrothermal treatment is consistent with previously published data [48], while the rod-like shape of particles is characteristic of α-MnO_2_ [49]. The SEM showed that the shape of particles was typical of α-MnO_2_ (Figure 1b), thus confirming the phase composition of the obtained powder, as well as indicating the absence of impurities of other phases.

The synthesised α-MnO_2_ powders were used to obtain composite elastomers, while vulcanisation of polydimethylsiloxane in the absence of an electric field and in an electric field of 2 kV/mm made it possible to obtain materials containing either stochastically or orderly distributed α-MnO_2_ particles (see Figure S1). The ordering of manganese dioxide particles in polydimethylsiloxane, which occurs as a result of the action of an electric field, due to particle polarisation, is in line with the authors’ previous results [36].

The results of the analysis of the degree of swelling degree for the obtained elastomers (Table 1) revealed that MnO_2_-0 and MnO_2_-E composites were characterised by similar values of the gel fraction and unvulcanised polymer content. However, the degree of crosslinking for the disordered elastomer cured in the absence of an electric field was significantly higher than for the ordered elastomer cured in an electric field and containing oriented α-MnO_2_ particles.

These data and a comparison with the results of the authors’ recent studies of the degree of crosslinking of electrorheological elastomers based on polydimethylsiloxane filled with highly dispersed amorphous titanium dioxide [50] demonstrate the significant effect of the filler on the degree of crosslinking in the polymer matrix. The vulcanisation of polydimethylsiloxane filled with titanium dioxide particles led to the production of elastomers characterised by a degree of crosslinking an order of magnitude higher than for polydimethylsiloxane filled with α-MnO_2_ nanoparticles [50]. In fact, the volume fraction of TiO_2_ in the polymer was almost twice as high (17 vol.%) as the volume fraction of α-MnO_2_ (9.4 vol.%). This difference was most likely due to the fact that hydrated titanium dioxide interacts with the components of the curing agent (aminopropyltriethoxysilane and tetraethoxysilane) to form Si–O–Ti bonds, (similar observations have been made earlier [51,52,53]), and thereby is chemically immobilised in the polymer matrix. Conversely, the crystalline α-MnO_2_ apparently is more likely to be physically immobilised in the matrix.

It should be emphasised that these observations of the effect of the type of filler on the degree of crosslinking in the polymer matrix are extremely important in the development of magneto- and electrorheological materials with desired properties, since the structure and composition of the composite have a significant effect on its dielectric behaviour.

**Figure 2:**
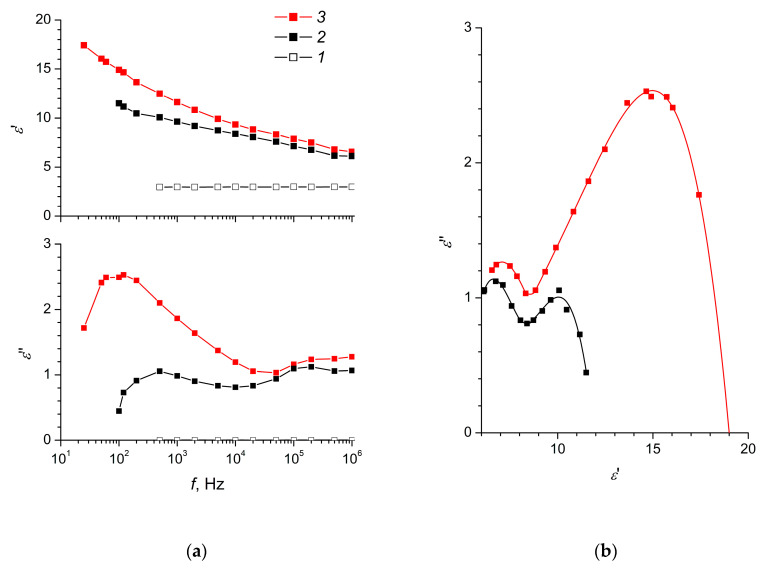
(**a**) The dielectric constants (*ε*’, *ε*”) as functions of frequency and (**b**) Cole–Cole diagrams for (*1*) unfilled elastomer, (*2*) MnO_2_-0 and (*3*) MnO_2_-E samples.

The dielectric spectra of the MnO_2_-0 and MnO_2_-E composite elastomers are shown in Figure 2.

**Figure 3:**
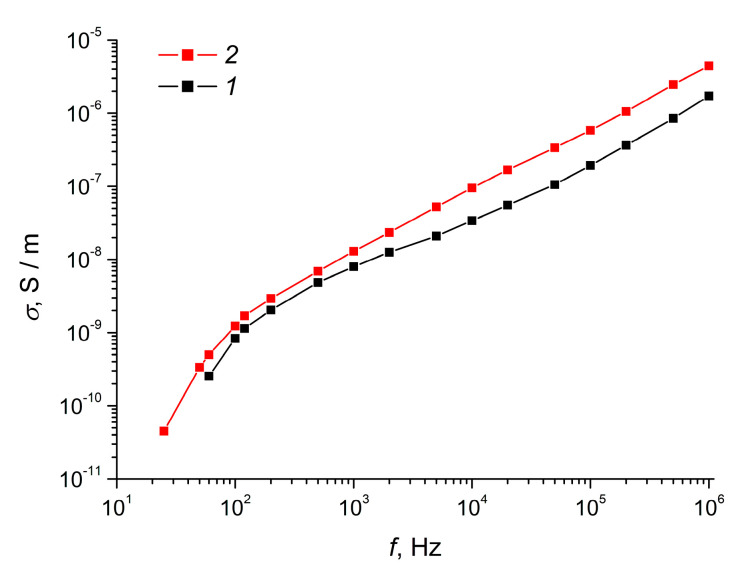
Conductivity of elastomeric composites filled with (*1*) MnO_2_-0 or (*2*) MnO_2_-E as a function of frequency.

From the data shown in Figure 2a, it follows that, for the obtained composite elastomers, the observed frequency dependence of *ε*” had pronounced resonance maxima in the low-frequency region and less pronounced resonance in the high-frequency region. In this case, the maxima for the ordered elastomer MnO_2_-E were characterised by a higher amplitude, demonstrating a more prominent interaction of the material with the electric field. The indicated maxima for both elastomers are asymmetric, while the nature of the asymmetry of the maxima is similar. Analysis of Cole–Cole diagrams [54] for both composite elastomers suggests that the low-frequency maximum for *ε*” is associated with the processes of the volume and surface polarisation of MnO_2_ nanoparticles. The decrease in the value of *ε*” with an increase in frequency to 10^3^–10^4^ Hz is associated with a delay in the reorientation of charges relative to the change in the electric field. The relaxation maximum in the high-frequency region can be associated with the formation of polarons and charge transfer between the dipoles of α-MnO_2_. This process reflects the frequency dependences of the conductivity of nanocomposites (Figure 3), typical for materials in which the classical hopping mechanism of charge transfer is realised [55]. In addition, the asymmetric appearance of Cole–Cole diagrams (Figure 2b) indicates the mobility of the polymer matrix at the molecular (polymer chain segments) and macroscopic (polymer globules) scales [56].

The frequency dependences of *ε*” (Figure 2) make it possible to estimate the characteristic relaxation times for both composite elastomers using the relation $\tau =\frac{1}{2\pi \omega }$ (*ω* is the angular frequency corresponding to the position of the relaxation maximum). The obtained estimates of the τ values for the disordered MnO_2_-0 were 3.2 × 10^−4^ s and 6.8 × 10^−7^ s, while for the ordered MnO_2_-E they were 1.6 × 10^−3^ s and 5.4 × 10^−7^ s, which corresponds to the characteristic times of the Maxwell–Wagner and ion-dipole relaxation processes. The indicated values also meet empirical criteria corresponding to high values of the electrorheological effect in electrorheological fluids [57,58].

From Figure 2a, it also follows that the dielectric constant of the ordered MnO_2_-E nanocomposite in the low-frequency region was significantly higher than the dielectric constant of the disordered MnO_2_-0 composite. These differences are associated with a significantly greater polarisation of mutually oriented nanoparticles of manganese dioxide in the MnO_2_-E elastomer. With increasing frequency, the values of dielectric constants of MnO_2_-0 and MnO_2_-E become closer to each other, thus the anisotropy of the structure of the composite elastomer barely affects the dielectric constant of the material in the high-frequency region.

Analysis of the set of dielectric parameters (Figure 2) and the conductivity (Figure 3) of elastomers filled with α-MnO_2_ nanoparticles allows us to draw a number of conclusions about the mechanisms of relaxation processes in these materials in a wide frequency range [59]. At a constant or low-frequency (10–10^5^ Hz) electric field in a composite consisting of a dielectric polymer matrix and a filler with relatively high conductivity, mobile charges are localised on the surface of filler particles, and the nature of polarisation processes is determined by the conductivity of the components [22]. In an alternating electric field in a frequency range of 10^4^–10^6^ Hz, the nature of polarisation processes in the system is determined not only by the conductivity of the components, but also by their dielectric characteristics. The conductivity of the ordered composite MnO_2_-E was higher in the entire frequency range compared to the disordered composite MnO_2_-0, which was apparently due to the formation of chain structures from filler particles in MnO_2_-E (Figure 3). At the same time, the described properties of the composites can be caused not only by the ordering of the filler particles in the MnO_2_-E composite, but also by the different degree of crosslinking of the elastomer and the presence of polymer molecules in the composite that are not linked into a common polymer network (see Table 1) [50].

**Figure 4:**
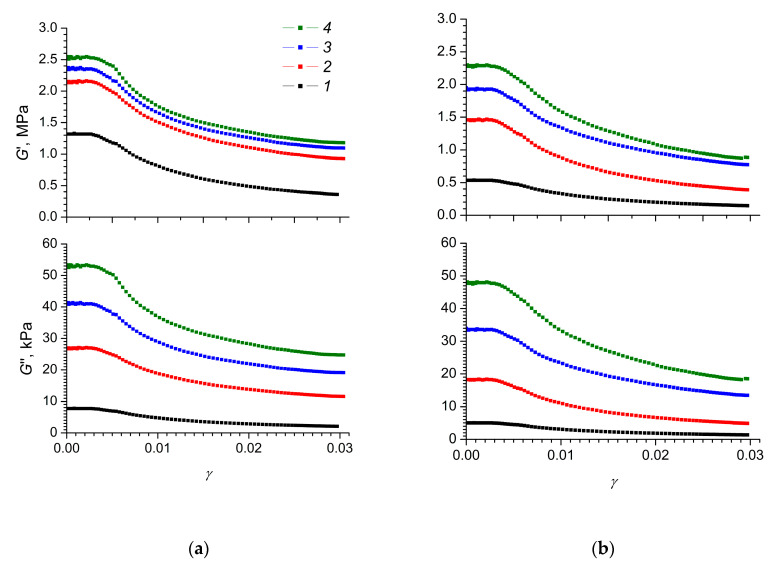
Dependences of storage (*G*′) and loss (*G*′′) moduli for (**a**) MnO_2_-0 and (**b**) MnO_2_-E composite elastomers on the degree of shear strain *γ* at various strengths of the electric field applied: *1*—0 kV/mm, *2*—0.4 kV/mm, *3*—1.2 kV/mm, *4*—2 kV/mm. Shear strain rate was 0.05 rad/s.

To quantify the magnitude of the electrorheological effect in composite elastomers we used the value of the shear elastic modulus G* [60]. G* is usually represented as a complex number G* = G′ + *i*G′′, where *G*′ is the storage modulus and *G*′′ is the loss modulus. The storage modulus *G*′ characterises the energy consumption of the elastic deformation and the loss modulus *G*′′ characterises the energy dissipation at viscous losses. Figure 4 shows the storage and loss moduli as functions of the degree of shear strain for the MnO_2_-0 and MnO_2_-E samples at different strengths of the applied electric field.

The values of the storage moduli for MnO_2_-0 and MnO_2_-E elastomers in the absence of an applied electric field, at a shear rate tending towards zero, were 1.32 and 0.52 MPa, respectively. A higher value of the storage modulus for a disordered composite elastomer MnO_2_-0 may indicate a more efficient reinforcement of the elastomer with chaotically oriented rod-like α-MnO_2_ nanoparticles. In contrast, the oriented arrangement of nanoparticles in the MnO_2_-E elastomer contributed to a reduction in its strength and resistance to a twisting force. Thus, our a priori hypothesis that polydimethylsiloxane containing oriented α-MnO_2_ rod-like particles will show higher rigidity was not confirmed.

On the other hand, a large value for the storage and loss moduli is typical of a disordered elastomer with a higher degree of cross-linking of the polymer matrix and hence lower elasticity (MnO_2_-0). Obviously, high values of the shear storage modulus narrow the range of variation of the elastic characteristics of the material in an electric field. At the same time, in the absence of an applied electric field, the elastomer must provide a sufficiently high elastic response to mechanical loads and must be able to withstand long-term loads, as these characteristics determine its properties as a structural material.

When an electric field was applied to the MnO_2_-0 and MnO_2_-E elastomers, there was a natural increase in the values of the storage and loss moduli (Figure 4), which was manifested as a pronounced electrorheological effect in these composite elastomers. Thus, the storage modulus of a disordered MnO_2_-0 elastomer in a 2.0 kV/mm electric field exceeded the analogous value for the same sample in the absence of an electric field by 1.2 MPa. For the ordered elastomer MnO_2_-E, a similar difference in the values of the storage modulus was 1.8 MPa.

Loss moduli of composite elastomers MnO_2_-0 and MnO_2_-E also depended markedly on the electric field strength (Figure 4). In particular, the loss modulus for a disordered MnO_2_-0 elastomer in the absence of an applied electric field was 8.5 kPa, while for the ordered MnO_2_-E it was 6.5 kPa. The application of an electric field with a strength of 2 kV/mm provided an increase in the loss modulus of the said composite elastomers up to 53 kPa (six times) and up to 48 kPa (seven times), respectively. At the same time, the flow parameters for both composite elastomers were quite close and the contribution of the loss modulus *G*′′ to the total value of G* was relatively small, amounting to a fairly small percentage. A small contribution of the loss modulus to the value of the shear modulus is characteristic of silicone elastomers [61,62].

When developing devices based on electrorheological elastomers, it is necessary to take into account the possible nonlinear dependence of their mechanical characteristics on the electric field strength and the degree of deformation. At a fixed value of the electric field strength, the elastomers obtained during the study demonstrated constant values of the storage and loss moduli up to the degree of deformation ~0.005. It is noteworthy that, in Figure 4, the width of the horizontal sections of the plots for both elastomer samples was approximately the same.

At significant deformations, the storage and loss moduli decreased, reaching nearly constant values at *γ* ≈ 0.3. Thus, under shear strain, including under an applied electric field, the elastomers obtained demonstrated the Payne effect [4,63]. The mechanism of this effect in composite elastomers is associated with the fact that the interparticle interactions make a significant contribution to the elasticity of the composite at low strain rates. Deformation of the composite elastomer leads to a decrease in interactions between filler particles, which in turn leads to a decrease in the elasticity of the elastomer.

For a quantitative comparison of the Payne effect in various materials one can use the Kraus model [64]:

(5) $$G^{\prime }\left(\gamma \right)=G_{\infty }^{\prime }+\frac{G_{0}^{\prime }-G_{\infty }^{\prime }}{1+{\left(\frac{\gamma }{\gamma_{0}}\right)}^{\beta }},$$

where $G^{\prime }\left(\gamma \right)$ is the storage modulus value at a given degree of deformation; $G_{0}^{\prime }$ and $G_{\infty }^{\prime }$—values of the storage modulus at zero and an infinitely large deformation, respectively, $\gamma_{0}$ is a characteristic deformation value corresponding to the breakage of a half of contacts between the filler particles; β is the parameter indicating shear stress sensitivity. Using Equation (5) we estimated $G_{0}^{\prime }$ and $G_{\infty }^{\prime }$ values which were further used to calculate the range of the storage modulus variation at various electric field strengths, $\eta_{p}^{\prime }=(G_{0}^{\prime }-G_{\infty }^{\prime })/G_{\infty }^{\prime }$, which characterizes the magnitude of the Payne effect. The $\eta_{p}^{\prime }$ values for MnO_2_-0 and MnO_2_-E elastomers in electric fields of various strengths are presented in Table 2.

The MnO_2_-0 and MnO_2_-E elastomers in the absence of an electric field exhibit a similarly high Payne effect (Table 2). For both elastomers, the application of an electric field leads to a decrease in the magnitude of the Payne effect. In this case, the application of an electric field to an elastomer with chaotically distributed rod-like α-MnO_2_ particles leads to a significantly greater decrease in the Payne effect than for the sample of elastomers with anisotropically oriented filler particles. It is worth mentioning that the magnitude of the Payne effect is nearly independent of the magnitude of the applied electric field.

The efficiency of the electrorheological elastomer can be estimated by comparing the values of the storage and loss moduli in the presence and absence of an electric field [6]. A similar approach to assessing the absolute magnitude of the magnetorheological effect by comparing the maximum value of the storage modulus achieved in a magnetic field and the storage modulus in the absence of a magnetic field has been described earlier [65]. For a quantitative description of the magnitude of the electrorheological effect, the use of a dimensionless ratio of the value of the measured rheological parameter in an electric field to its value, in the absence of an electric field, was proposed [31]. This ratio can be used as a criterion for assessing the efficiency of converting electrical energy into mechanical energy. In this regard, to analyse the electrorheological effect in composite elastomers, we used the ratio [31]:

(6) $$\eta_{E}^{\prime }=\frac{G_{E}^{\prime }}{G_{0}^{\prime }},$$

where $G_{E}$—the storage or loss modulus of elastomers in an electric field; $G_{0}$—the storage or loss modulus of elastomers in the absence of an electric field.

**Figure 5:**
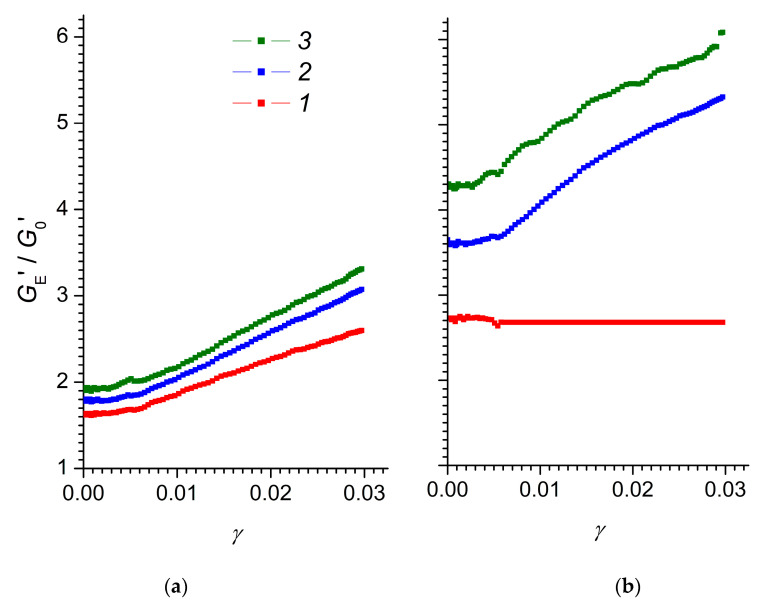
The storage modulus magnitude of the electrorheological effect for elastomers (**a**) MnO_2_-0 and (**b**) MnO_2_-E as a function of the degree of deformation in electric fields with strengths *1*—0.4, *2*—1.2, *3*—2.0 kV/mm.

Figure 5 shows magnitudes of the electrorheological effect $\eta_{E}^{\prime }$ of elastomers in electric fields with strengths ranging from 0.4 to 2 kV/mm, as functions of the degree of deformation.

It should be noted that, until now, the electrorheological efficiency of elastomers has been evaluated for only one fixed value of the shear rate [6]. At the same time, the analysis of the dependences of electrorheological efficiency on the degree of deformation makes it possible to reveal important features of the interaction of the system with the electric field.

The dependence of the magnitude of the electrorheological effect on the degree of deformation for elastomers with chaotically, and orderly, distributed filler particles was significantly different. In the region of relatively small deformations (up to *γ* ≈ 0.005), there was a section in which the magnitude of the electrorheological effect was virtually independent of the degree of deformation. For a disordered elastomer MnO_2_-0, an increase in the electrorheological effect was observed for the entire range of electric field strengths, with an increase in the degree of deformation. Conversely, for the ordered elastomer MnO_2_-E, at low electric field strengths (0.4 kV/mm), the electrorheological effect did not depend on the degree of deformation and only in strong electric fields (1.2 and 2.0 kV/mm) was there an increase in the electrorheological effect with an increase in the degree of deformation. It is obvious that the electrorheological effect in composite elastomers at small deformations was due to electrostatic interaction between the filler particles, which contributed to an increase in the elasticity of the polymer matrix. With increasing deformation, the elasticity of the polymer matrix decreased due to the stretching of polymer molecules and the partial rupture of filler polymer-nanoparticle bonds and weakening of the elasticity of the composite.

For practical purposes, the region of deformations in which the loss of elasticity of the elastomer was not observed is of most interest. Since, for the composites obtained, this region was located below ~0.005 degrees of deformation, their electrorheological efficiency should be evaluated either at low, or at zero degrees of deformation, extrapolating the experimental data to a zero value of *γ*. Thus, for the disordered MnO_2_-0 elastomer, the electrorheological effect $\eta_{E}^{\prime }$ at zero strain increases from 1.6 to 1.9, with an increase in the electric field strength from 0.4 to 2.0 kV/mm. Under analogous conditions, the electrorheological effect for the ordered MnO_2_-E elastomer increases from 2.7 to 4.3.

The results obtained demonstrate the possibility of creating a highly efficient electrorheological elastomer that changes elastic properties by up to 4.3 times in the range of field strengths up to 2 kV/mm.

## 4. Conclusions

For the first time, composite elastomers have been obtained based on vulcanised silicone rubber filled with rod-like α-MnO_2_ nanoparticles, with either stochastic or ordered distribution of nanoparticles. The degree of crosslinking of elastomers was shown to be higher for an elastomer with a stochastic distribution of nanoparticles cured in the absence of an electric field. The obtained composite elastomers are characterised by the presence of two types of polarization—interphase and ion-dipole. A detailed analysis of the electrorheological effect, namely the influence of the electric field on the storage and loss moduli during shear deformation, revealed the manifestation of the Payne effect in composite elastomers. It has been shown, for the first time, that the application of an electric field with a strength of at least 0.4 kV/mm leads to a decrease in the Payne effect in composite elastomers. For a disordered composite cured in the absence of an electric field, the storage modulus value increased by 1.9 times in the 2 kV/mm electric field. For an ordered elastomer cured in an electric field, the storage modulus value increased by 4.3 times.

## Figures and Tables

**Table 1:** Results of the analysis of the degree of crosslinking for MnO_2_-0 and MnO_2_-E samples.

Sample	Swelling Ratio *β*, %	Gel Fraction GF, %	Uncured Polymer Content $\omega_{c},\;\%$	Degree of Crosslinking ν, mol/cm^3^
MnO_2_-0	118	97.5	2.4	7.6 × 10^−4^
MnO_2_-E	188	96.9	3.0	3.3 × 10^−4^

**Table 2:** The values of $\eta_{p}^{\prime }$ in MnO_2_-0 and MnO_2_-E elastomers as a function of the electric field strength.

Sample	E, kV/mm			
	0.0	0.4	1.2	2.0
MnO_2_-0	4.6	1.5	1.5	1.4
MnO_2_-E	4.7	3.8	3.7	3.8

## Supplementary Materials

The following are available online at https://www.mdpi.com/2073-4360/12/12/2810/s1, Figure S1: The appearance of α-MnO_2_ suspension at 40× magnification in uncured polydimethylsiloxane between the electrodes (**a**) in the absence and (**b**) in the presence of an electric field. The interelectrode gap was 1 mm.
