# Supplementary material for: Polydimethylsiloxane Elastomers Filled with Rod-Like α-MnO2 Nanoparticles: An Interplay of Structure and Electrorheological Performance
Source: Polymers (Basel). 2020 Nov 27;12(12):2810. doi: 10.3390/polym12122810 (PMC7759881; doi:10.3390/polym12122810)
Supplement: Supplementary file 1 [file polymers-12-02810-s001.pdf]

polymers

Electronic Supplementary Information

Polydimethylsiloxane elastomers filled with rod-like α-MnO_2 nanoparticles: An interplay of structure and electrorheological performance

Alexander V. Agafonov[1,2], Anton S. Kraev[1], Anastasia A. Egorova[3], Alexander E. Baranchikov[3], Sergey A. Kozyukhin[3], Vladimir K. Ivanov[3,4]*

1 Krestov Institute of Solution Chemistry of the Russian Academy of Sciences, Ivanovo, 153045, Russia; ava@isc-ras.ru
2 National Research Tomsk State University, Tomsk, 634050, Russia
3 Kurnakov Institute of General and Inorganic Chemistry of the Russian Academy of Sciences, Moscow, 119991, Russia; a.baranchikov@yandex.ru
4 National Research University Higher School of Economics, Moscow, 101000, Russia
* Correspondence: van@igic.ras.ru

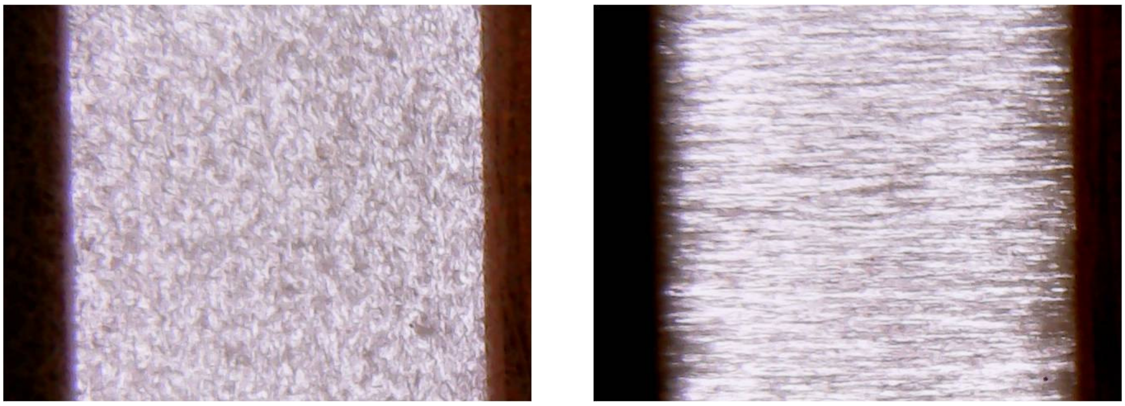

(**a**) (**b**)

Figure S1. The appearance of α-MnO_2 suspension at 40× magnification in uncured polydimethylsiloxane between the electrodes (**a**) in the absence and (**b**) in the presence of an electric field. The interelectrode gap was 1 mm.
